# XELOX *vs* FOLFOX-4 as first-line therapy for metastatic colorectal cancer: NO16966 updated results

**DOI:** 10.1038/bjc.2011.201

**Published:** 2011-06-14

**Authors:** J Cassidy, S Clarke, E Díaz-Rubio, W Scheithauer, A Figer, R Wong, S Koski, K Rittweger, F Gilberg, L Saltz

**Affiliations:** 1 Institute of Cancer Sciences, Beatson Oncology Centre, 1053 Great Western Road, Glasgow G12 0YN, UK; 2 Sydney Medical School, University of Sydney and Sydney Cancer Centre, Royal North Shore Hospital, New South Wales 2006, Australia; 3 Servicio de Oncologia Medica, Hospital Clínico San Carlos, Profesor Martin Lagos S/N, Madrid 28040, Spain; 4 Department of Internal Medicine, Vienna University Medical School, Spitalgasse 23, 1090 Vienna, Austria; 5 Department of Medical Oncology, Tel Aviv Sourasky Medical Center, 6 Weizmann Street, Tel Aviv 64239, Israel; 6 Department of Medical Oncology, Cancer Care Manitoba, St Boniface General Hospital, 409 Taché Avenue, Winnipeg, Manitoba R2N 2A6, Canada; 7 Department of Oncology, Cross Cancer Institute, 11560 University Avenue Nw, Edmonton, Alberta T6G 1Z2, Canada; 8 Department of Clinical Science, Hoffmann-La Roche Inc., 340 Kingsland Street, Nutley, NJ 07110, USA; 9 Department of Statistics, F. Hoffmann–La Roche, Grenzacherstrasse 124, 4070 Basel, Switzerland; 10 Department of Medicine, Memorial Sloan Kettering Cancer Center, 1275 York Avenue, New York, NY 10065, USA

**Keywords:** 5-fluorouracil/folinic acid, capecitabine, colorectal cancer, overall survival, oxaliplatin

## Abstract

**Background:**

We report updated overall survival (OS) data from study NO16966, which compared capecitabine plus oxaliplatin (XELOX) *vs* 5-fluorouracil/folinic acid plus oxaliplatin (FOLFOX4) as first-line therapy in metastatic colorectal cancer.

**Methods:**

NO16966 was a randomised, two-arm, non-inferiority, phase III comparison of XELOX *vs* FOLFOX4, which was subsequently amended to a 2 × 2 factorial design with further randomisation to bevacizumab or placebo. A planned follow-up exploratory analysis of OS was performed.

**Results:**

The intent-to-treat (ITT) population comprised 2034 patients (two-arm portion, *n*=634; 2 × 2 factorial portion, *n*=1400). For the whole NO16966 study population, median OS was 19.8 months in the pooled XELOX/XELOX-placebo/XELOX-bevacizumab arms *vs* 19.5 months in the pooled FOLFOX4/FOLFOX4-placebo/FOLFOX4-bevacizumab arms (hazard ratio 0.95 (97.5% CI 0.85–1.06)). In the pooled XELOX/XELOX-placebo arms, median OS was 19.0 *vs* 18.9 months in the pooled FOLFOX4/FOLFOX4-placebo arms (hazard ratio 0.95 (97.5% CI 0.83–1.09)). FOLFOX4 was associated with more grade 3/4 neutropenia/granulocytopenia and febrile neutropenia than XELOX, and XELOX with more grade 3 diarrhoea and grade 3 hand-foot syndrome than FOLFOX4.

**Conclusion:**

Updated survival data from study NO16966 show that XELOX is similar to FOLFOX4, confirming the primary analysis of progression-free survival. XELOX can be considered as a routine first-line treatment option for patients with metastatic colorectal cancer.

Capecitabine (Xeloda; F. Hoffmann–La Roche, Basel, Switzerland) is an oral fluoropyrimidine with similar efficacy to bolus 5-fluorouracil/folinic acid (5-FU/FA) in the first-line treatment of metastatic colorectal cancer (Hoff *et al*, 2001; van Cutsem *et al*, 2001) and as adjuvant therapy for stage III colon cancer (Twelves *et al*, 2005). The efficacy of capecitabine and a 3-weekly dose of oxaliplatin (XELOX regimen) is also non-inferior to 5-FU/FA plus oxaliplatin (FOLFOX4 or FOLFOX6) in the first- and second-line treatment of patients with metastatic colorectal cancer (Rothenberg *et al*, 2008; Cassidy *et al*, 2008a; Ducreux *et al*, 2011).

The largest of the recent trials evaluating XELOX, NO16966 (XELOX-1), started as a two-arm, randomised, non-inferiority, phase III study designed to compare XELOX with FOLFOX4 as first-line treatment for metastatic colorectal cancer. The protocol was subsequently amended to include the further addition of bevacizumab or placebo using a 2 × 2 factorial design. The study had two co-primary objectives: (1) to evaluate the non-inferiority of XELOX ± bevacizumab *vs* FOLFOX4 ± bevacizumab; and (2) to evaluate the superiority of bevacizumab *vs* placebo when combined with oxaliplatin-based chemotherapy (i.e., XELOX or FOLFOX4). The results pertaining to both objectives have been previously published (Saltz *et al*, 2008; Cassidy *et al*, 2008a). The present paper describes an updated analysis of overall survival (OS) for the comparison of XELOX/XELOX-placebo/XELOX-bevacizumab *vs* FOLFOX4/FOLFOX4-placebo/FOLFOX4-bevacizumab from study NO16966.

## Patients and methods

The methods of this trial have been described in detail previously (Saltz *et al*, 2008; Cassidy *et al*, 2008a). The study was performed in accordance with the Declaration of Helsinki and Good Clinical Practice Guidelines. Written informed consent was obtained from all patients participating in the study. Approval of the protocol was obtained from an independent ethics committee or institutional review board of each site.

### Patient population

Patients ⩾18 years of age with histologically confirmed, unresectable metastatic colorectal cancer (⩾1 unidimensionally measurable lesion), an ECOG performance status of ⩽1 and a life expectancy of >3 months were eligible. No previous systemic therapy for metastatic disease or previous oxaliplatin or bevacizumab were allowed. Patients were required to have adequate haematologic/clotting, hepatic and renal function. Patients were excluded if they had any of the following: clinically significant cardiovascular disease; clinically detectable ascites; use of full-dose anticoagulants or thrombolytics; known CNS metastases; serious non-healing wound, ulcer or bone fracture; known bleeding diathesis or coagulopathy; and proteinuria ⩾500 mg per 24 h.

### Treatment plan

Patients were randomly assigned to treatment using an interactive voice response system. Randomisation was stratified by region, ECOG performance status, liver as a metastatic site, number of metastatic sites (organs) and alkaline phosphatase level.

XELOX consisted of a 2-h intravenous infusion of oxaliplatin 130 mg m^–2^ on day 1 plus oral capecitabine 1000 mg m^–2^ twice daily for 2 weeks as a 3-week cycle. The first dose of capecitabine was given in the evening of day 1 and the last dose on the morning of day 15. The FOLFOX4 regimen has been previously described (de Gramont *et al*, 2000). After amendment of the study protocol, bevacizumab (7.5 mg kg^–1^ every third week) or placebo was added to XELOX, and bevacizumab (5 mg kg^–1^ every second week) or placebo to FOLFOX4. Bevacizumab or placebo was given as a 30- to 90-min intravenous infusion on day 1 of each cycle before oxaliplatin.

Treatment was continued until disease progression or for 48 weeks, whichever came first (study treatment phase). Patients who completed the 48-week treatment phase without disease progression were eligible to continue treatment until progression (post-study treatment phase). Patients whose tumour became operable, and for whom resection was performed, were allowed to enter the post-study treatment phase.

### Assessments

Tumour assessments (CT scan, MRI) were performed within 28 days before starting study treatment and repeated after every two XELOX cycles and every three FOLFOX4 cycles (i.e., every sixth week in both arms), and at the end of treatment. After completion of study treatment, patients were followed every 3 months until disease progression and/or death.

Patients were evaluated for adverse events during therapy and until 28 days after the last study drug dose. Adverse events were graded according to National Cancer Institute Common Toxicity Criteria (NCI-CTC), version 3. Predefined adverse events of special interest for chemotherapy were: grade 3/4 neutropenia/granulocytopenia; grade 3/4 neurosensory toxicity; grade 3/4 diarrhoea; grade 3/4 vomiting/nausea; grade 3/4 stomatitis and grade 3 hand-foot syndrome.

### Statistical analysis

The intent-to-treat (ITT) patient population included all patients who underwent randomisation and signed the informed consent form. The eligible patient population (EPP) was the ITT population minus patients who did not receive at least one dose of study drug, and those patients who violated major protocol inclusion/exclusion criteria. As the results for the EPP population were the same as for the ITT population, ITT data only will be presented in this paper. The safety population included all patients receiving at least one dose of study drug.

Overall survival was defined as the time from the date of randomisation to the date of death. Patients who were not reported as having died at the time of the analysis were censored using the date they were last known to be alive. Overall survival was analysed using a Cox model and presented as Kaplan–Meier estimates with hazard ratios (HRs) and 97.5% confidence intervals (CIs).

The primary analysis of NO16966 was event driven and was performed on 31 January 2006 when 1200 progression-free survival events had occurred in the EPP; this approach ensured 90% power at an *α* level of 2.5 (Saltz *et al*, 2008). A further planned follow-up analysis of OS was performed at the time of the 4-month safety update.

As the study was not powered for formal testing of non-inferiority for OS, the OS analysis is exploratory and the results described by Kaplan–Meier estimates with HRs and 97.5% CIs. An additional exploratory analysis of OS was performed to control for any possible crossover effects of FOLFOX in patients who received XELOX as their first-line regimen. In this analysis, patients in the XELOX arms who received FOLFOX4 or similar regimen as second-line therapy were censored.

## Results

### Patient population

**Figure 1:**
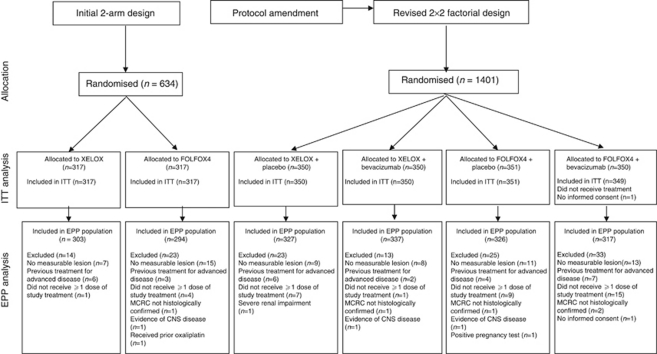
The CONSORT flowchart.

Between July 2003 and May 2004, 634 patients were randomised in the two-arm portion of the study. Between February 2004 and February 2005, a further 1400 patients were randomised in the 2 × 2 factorial part of the study. Overall, 2034 patients made up the ITT population (Figure 1). The baseline demographic and clinical characteristics were well balanced between treatment arms (Table 1).

### Treatment exposure and second-line therapy

The median dose intensities (ratio of dose received to dose planned) of 5-FU, capecitabine, oxaliplatin and bevacizumab were ⩾0.89 in all treatment arms. The median number of cycles administered was 11 (range 1–24) in the FOLFOX4/FOLFOX4-placebo group, 12 (range 1–25) in the FOLFOX4-bevacizumab group, 7 (range 1–18) in the XELOX/XELOX-placebo group and 8 (range 1–17) in the XELOX-bevacizumab group.

There were no major imbalances between the treatment groups with respect to the use of second-line therapy: XELOX-containing arms (65%) and FOLFOX4-containing arms (70%). The agents most commonly used were: irinotecan (56% with FOLFOX4 *vs* 53% with XELOX); 5-FU (41 *vs* 34%); capecitabine (19 *vs* 14%); cetuximab (20 *vs* 18%); and bevacizumab (10 *vs* 10%).

### Overall survival

**Figure 2:**
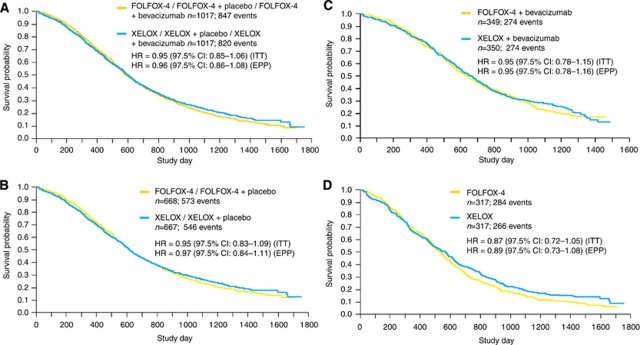
Overall survival for FOLFOX4/FOLFOX4-placebo/FOLFOX4-bevacizumab *vs* XELOX/XELOX-placebo/XELOX-bevacizumab (**A**), FOLFOX4/FOLFOX4-placebo *vs* XELOX/XELOX-placebo (**B**), FOLFOX4-bevacizumab *vs* XELOX-bevacizumab (**C**) and FOLFOX4 *vs* XELOX (**D**) (ITT population).

The OS data as at 31 July 2008 in the ITT population are shown in Table 2. The corresponding Kaplan–Meier curves for OS are shown in Figure 2.

For the whole NO16966 study population, median OS was 19.8 months in the pooled XELOX/XELOX-placebo/XELOX-bevacizumab arms *vs* 19.5 months in the pooled FOLFOX4/FOLFOX4-placebo/FOLFOX4-bevacizumab arms, with a corresponding HR of 0.95 (97.5% CI 0.85–1.06).

In the pooled XELOX/XELOX-placebo arms, median OS was 19.0 *vs* 18.9 months in the pooled FOLFOX4/FOLFOX4-placebo arms, with a corresponding HR of 0.95 (97.5% CI 0.83–1.09).

In the XELOX-bevacizumab arm, median OS was 21.6 *vs* 21.0 months in the FOLFOX4-bevacizumab arm, with a corresponding HR of 0.95 (97.5% CI 0.78–1.15).

In the XELOX arm, median OS was 18.8 *vs* 17.7 months in the FOLFOX4 arm, with a corresponding HR of 0.87 (97.5% CI 0.72–1.05). FOLFOX4 or a similar regimen was given to 8% of patients in the pooled XELOX arms as second-line therapy (XELOX, *n*=15, XELOX-placebo, *n*=38, XELOX-bevacizumab, *n*=29). After censoring these patients, the median OS was 18.9 months in the pooled XELOX/XELOX-placebo arms and 18.9 months in the pooled FOLFOX4/FOLFOX4-placebo arms, with a corresponding HR of 0.94 (97.5% CI 0.82–1.08), and 21.6 months in the XELOX-bevacizumab arm and 21.0 months in the FOLFOX4-bevacizumab arm (HR=0.93; 97.5% CI 0.76–1.13).

### Safety

For the updated safety assessment of XELOX *vs* FOLFOX4, patients in the pooled XELOX/XELOX-placebo (*n*=655) and pooled FOLFOX-4/FOLFOX4-placebo (*n*=648) arms were compared. The updated safety analysis showed that little had changed since the previous analysis (Cassidy *et al*, 2008a). Predefined adverse events of special interest and key events pooled by body system are presented in Table 3.

In general, XELOX and FOLFOX4 had a similar profile of adverse events. The most common adverse events were gastrointestinal (i.e., diarrhoea, nausea, vomiting and stomatitis) and neurosensory toxicities (i.e., paraesthesia and peripheral neuropathy). However, there were differences between the two regimens in the rates at which key events occurred. FOLFOX4/FOLFOX4-placebo was associated with more grade 3/4 neutropenia/granulocytopenia (44%) and febrile neutropenia (5%) than XELOX/XELOX-placebo (7 and <1%, respectively). Conversely, XELOX/XELOX-placebo was associated with more hand-foot syndrome (all-grade, 31 *vs* 11% grade 3, 6 *vs* 1%) and diarrhoea (all-grade, 66 *vs* 61% grade 3/4, 20 *vs* 11%) than FOLFOX4/FOLFOX4-placebo, although the rate of grade 4 diarrhoea was 1% with both regimens. Rates of grade 3/4 neurosensory toxicity were similar with both regimens (17%). Cardiac disorders were reported in 6 (1%) XELOX/XELOX-placebo recipients and 9 (1%) FOLFOX-4/FOLFOX4-placebo recipients. The addition of bevacizumab did not alter the similarities and differences in safety profiles between XELOX and FOLFOX4 (Table 4).

Treatment-related mortality up to 28 days after the last treatment dose was documented in 11 (1.7%) FOLFOX4/FOLFOX4-placebo patients and in 15 (2.3%) XELOX/XELOX-placebo patients. The respective 60-day all-cause mortality rates were 2.3% (*n*=15) and 3.4% (*n*=22).

## Discussion

The primary efficacy analysis of study NO16966 showed that XELOX is non-inferior to FOLFOX4 in terms of progression-free survival, OS and overall response rate in the first-line treatment of patients with metastatic colorectal cancer (Cassidy *et al*, 2008a). This updated analysis of OS again demonstrates that XELOX and FOLFOX4 have similar efficacy and supports the primary efficacy findings. It is also notable that both XELOX and FOLFOX4 were similar in terms of OS after the addition of bevacizumab.

Overall survival is the most clinically meaningful and objective measure of efficacy in patients with cancer. However, potential differences between study treatments can be masked by second-line and later lines of chemotherapy when this end point is used (Di Leo *et al*, 2004). In study NO16966, there were no restrictions regarding crossover or salvage therapies after the completion of study treatment. It is therefore possible that crossover to the alternate study treatment was a confounding factor in the present analysis. To allow for this, we performed a separate analysis in which all patients randomised to XELOX and who received FOLFOX as second-line therapy were censored. The results were consistent with those obtained in the ITT population and again support the similar efficacy of XELOX *vs* FOLFOX4.

The question of whether or not capecitabine is non-inferior to 5-FU/FA when given in combination with oxaliplatin in metastatic colorectal cancer has now been addressed in six different randomised phase III trials (Díaz-Rubio *et al*, 2007; Porschen *et al*, 2007; Rothenberg *et al*, 2008; Cassidy *et al*, 2008a; Comella *et al*, 2009; Ducreux *et al*, 2011), of which NO16966 is the largest. The other five studies, which involved 300–600 patients each, were largely supportive of NO16966. In three of the studies, the efficacy of XELOX or OXXEL was shown to be similar to that of 5-FU/FA-oxaliplatin regimens (Rothenberg *et al*, 2008; Comella *et al*, 2009; Ducreux *et al*, 2011), whereas the remaining two were inconclusive with regard to non-inferiority (Díaz-Rubio *et al*, 2007; Porschen *et al*, 2007). Since the completion of the phase III trials, three separate meta-analyses of relevant studies comparing capecitabine or 5-FU/FA plus oxaliplatin in patients with metastatic colorectal cancer have been performed (Arkenau *et al*, 2008; Cassidy *et al*, 2008b; Cuppone *et al*, 2008). Even though each meta-analysis included a different selection of phase II and III studies, the outcomes were very similar with respect to both progression-free survival (HR/relative risk 0.98–1.04) and OS (1.02–1.04). Thus, there is now strong evidence to support the non-inferiority of capecitabine when used in combination with oxaliplatin *vs* infusional 5-FU-based oxaliplatin regimens in the treatment of patients with metastatic colorectal cancer, both in the first- and second-line settings.

It is therefore likely that other considerations, such as tolerability profile, convenience, patient preference and cost, will assume greater importance when selecting the fluoropyrimidine backbone of a chemotherapy regimen. With regard to tolerability, both XELOX and FOLFOX have a similar profile of adverse events, but XELOX is associated with more grade 3 diarrhoea and hand-foot syndrome, whereas FOLFOX is associated with more grade 3/4 neutropenia and febrile neutropenia (Rothenberg *et al*, 2008; Ducreux *et al*, 2011). This is supported by the updated safety data from NO16966 in the present paper.

In terms of convenience, XELOX requires fewer planned office visits than the FOLFOX regimens because oxaliplatin is administered every 3 weeks (rather than every 2 weeks) and because capecitabine is taken orally. This is supported by resource use data from NO16966, which showed that the need for drug administration visits, central venous access and patient travel and time were reduced with XELOX *vs* FOLFOX4 (Scheithauer *et al*, 2007). When costs were assigned to these data, the total direct costs of both regimens were similar, whereas the indirect costs of XELOX were considerably less than those of FOLFOX4 (Garrison *et al*, 2007). Similar observations were made in a cost comparison of capecitabine ± oxaliplatin *vs* 5-FU ± oxaliplatin based on a retrospective analysis of a US medical claims database (Chu *et al*, 2009). Modified FOLFOX regimens, which involve a single 46- to 48-h infusion of 5-FU, are likely to be less costly than unmodified FOLFOX regimens (Garrison *et al*, 2007), although a complete assessment *vs* XELOX has yet to be performed.

In conclusion, updated survival data from study NO16966 show that XELOX is similar to FOLFOX4, confirming the primary analysis of progression-free survival. XELOX can be considered as a routine first-line treatment option for patients with metastatic colorectal cancer.

## Figures and Tables

**Table 1:** Baseline patient characteristics (ITT population)

	**FOLFOX4 (*n*=317)**		**FOLFOX4- placebo (*n*=351)**		**FOLFOX4- bevacizumab (*n*=349)**		**XELOX (*n*=317)**		**XELOX- placebo (*n*=350)**		**XELOX- bevacizumab (*n*=350)**	
**Characteristic**	**No.**	**%**	**No.**	**%**	**No.**	**%**	**No.**	**%**	**No.**	**%**	**No.**	**%**
*Gender*												
Male	204	64	186	53	205	59	194	61	205	59	213	61
Female	113	36	165	47	144	41	123	39	145	41	137	39
												
*Age, years*												
Median	62		60		60		61		61		61	
Range	24–83		26–83		19–82		24–84		18–83		18–86	
												
*ECOG performance status*												
0	163	51	211	60	198	57	160	50	207	59	207	59
1	154	49	138	40	147	43	157	50	143	41	142	41
2	0	0	0	0	0	0	0	0	0	0	1	<1
												
*Primary tumour site*												
Colorectal	17	5	25	7	28	8	30	9	30	9	32	9
Colon	200	63	232	66	223	64	204	64	233	67	236	67
Rectal	100	32	94	27	98	28	83	26	87	25	82	23
												
*Stage at first diagnosis*												
Local regional	144	45	141	40	128	37	133	42	138	39	122	35
Metastatic	173	55	210	60	221	63	184	58	212	61	228	65
												
*Number of metastatic sites*												
0	1	0.3	1	0.3	1	0.3	0	0	0	0	0	0
1	118	37.2	142	40.5	150	43.0	127	40.1	155	44.3	134	38.3
2	121	38.2	122	34.8	132	37.8	106	33.4	112	32.0	121	34.6
3	47	14.8	65	18.5	44	12.6	55	17.4	58	16.6	64	18.3
⩾4	30	9.5	21	6.0	22	6.3	29	9.1	25	7.1	31	8.9
												
*Alkaline phosphatase*												
Abnormal	135	43	147	42	146	42	132	42	149	43	156	45
Normal	182	57	201	58	199	58	183	58	200	57	191	55
												
*Previous adjuvant therapy*												
No	234	74	266	76	261	75	229	72	259	74	274	78
Yes	83	26	85	24	88	25	88	28	91	26	76	22

Abbreviations: ITT=intent-to-treat; ECOG=Eastern Cooperative Oncology Group; FOLFOX4=infused fluorouracil, folinic acid and oxaliplatin; XELOX=capecitabine and oxaliplatin.

**Table 2:** Overall survival by treatment subgroup (ITT population)

**Treatment subgroup comparison**	**No. of events**	**Median time to event (months)**	**Hazard ratio (97.5% CI)**
FOLFOX4/FOLFOX4-placebo/FOLFOX4-bevacizumab	847	19.5	0.95 (0.85–1.06)
XELOX/XELOX-placebo/XELOX-bevacizumab	820	19.8	
FOLFOX4/FOLFOX4-placebo	573	18.9	0.95 (0.83–1.09)
XELOX/XELOX-placebo	546	19.0	
FOLFOX4-bevacizumab	274	21.0	0.95 (0.78–1.15)
XELOX-bevacizumab	274	21.6	
FOLFOX4	284	17.7	0.87 (0.72–1.05)
XELOX	266	18.8	

Abbreviations: ITT=intent-to-treat; CI=confidence interval; FOLFOX4=infused fluorouracil, folinic acid and oxaliplatin; XELOX=capecitabine and oxaliplatin.

**Table 3:** Adverse events of special interest to chemotherapy and key events pooled by body system (treatment-related and unrelated)

	**FOLFOX4/FOLFOX4-placebo (*n*=648)**				**XELOX/ XELOX-placebo (*n*=655)**			
	**All-grade**		**Grade 3/4**		**All-grade**		**Grade 3/4**	
	**No.**	**%**	**No.**	**%**	**No.**	**%**	**No.**	**%**
All events	644	99	506	78	649	99	468	72
								
*Body system*								
Gastrointestinal disorders	603	93	167	26	606	93	216	33
Blood/lymphatic disorders	448	69	318	49	312	48	104	16
Infections/infestations	292	45	66	10	210	32	45	7
								
*Events of special interest*								
Neurosensory toxicity^a^	515	80	107	17	534	82	114	17
Diarrhoea	394	61	74	11	429	66	133	20
Nausea/vomiting	452	70	47	7	464	71	52	8
Stomatitis	242	37	13	2	140	21	8	1
Neutropenia/granulocytopenia	379	59	282	44	180	28	46	7
Febrile neutropenia	—	—	31	5	—	—	6	<1
Hand-foot syndrome	70	11	8^b^	1^b^	201	31	40^b^	6^b^

Abbreviations: FOLFOX4=infused 5-fluorouracil, folinic acid and oxaliplatin; XELOX=capecitabine and oxaliplatin.

a Pooled term that includes burning sensation, dysaesthesia, hyper or hypoaesthesia, neuropathic pain, neuropathy, peripheral neuropathy, neurotoxicity, paraesthesia, peripheral (sensi)motor neuropathy, (chronic) polyneuropathy, sensory disturbance or loss, skin burning sensation, temperature intolerance, neuralgia, peroneal nerve palsy, autonomic neuropathy.

b Grade 3 events only.

**Table 4:** Adverse events of special interest to chemotherapy and key events pooled by body system (treatment-related and unrelated)

	**FOLFOX4-bevacizumab (*n*=342)**				**XELOX-bevacizumab (*n*=353)**			
	**All-grade**		**Grade 3/4**		**All-grade**		**Grade 3/4**	
	**No.**	**%**	**No.**	**%**	**No.**	**%**	**No.**	**%**
All events	340	99	289	85	351	99	266	75
								
*Body system*								
Gastrointestinal disorders	320	94	94	28	325	92	132	37
Blood/lymphatic disorders	229	67	159	47	125	35	44	13
Infections/infestations	164	42	31	9	137	39	21	6
								
*Events of special interest*								
Neurosensory toxicity^a^	281	82	61	18	296	84	64	18
Diarrhoea	219	64	44	13	224	64	77	22
Nausea/vomiting	235	69	25	7	252	71	38	11
Stomatitis	141	41	12	4	102	29	7	2
Neutropenia/granulocytopenia	189	55	138	40	70	20	25	7
Febrile neutropenia	—	—	15	4	—	—	4	1
Hand-foot syndrome	47	14	6^b^	2^b^	141	40	42^b^	12^b^

Abbreviations: FOLFOX4=infused 5-fluorouracil, folinic acid and oxaliplatin; XELOX=capecitabine and oxaliplatin.

a Pooled term that includes burning sensation, dysaesthesia, hyper or hypoaesthesia, neuropathic pain, neuropathy, peripheral neuropathy, neurotoxicity, paraesthesia, peripheral (sensi)motor neuropathy, (chronic) polyneuropathy, sensory disturbance or loss, skin burning sensation, temperature intolerance, neuralgia, peroneal nerve palsy, autonomic neuropathy.

b Grade 3 events only.

## References

[bib1] Arkenau HT, Arnold D, Cassidy J, Diaz-Rubio E, Douillard JY, Hochster H, Martoni A, Grothey A, Hinke A, Schmiegel W, Schmoll HJ, Porschen R (2008) Efficacy of oxaliplatin plus capecitabine or infusional fluorouracil/leucovorin in patients with metastatic colorectal cancer: a pooled analysis of randomized trials. J Clin Oncol 26: 5910–5917 19018087 10.1200/JCO.2008.16.7759

[bib2] Cassidy J, Clarke S, Diaz Rubio E, Scheithauer W, Figer A, Wong R, Koski S, Lichinitser M, Yang TS, Rivera F, Couture F, Sirzén F, Saltz L (2008a) A randomized phase III study of capecitabine plus oxaliplatin (XELOX) versus fluorouracil/folinic acid plus oxaliplatin (FOLFOX-4) as first-line therapy for metastatic colorectal cancer. J Clin Oncol 26: 2006–2012 18421053 10.1200/JCO.2007.14.9898

[bib3] Cassidy J, Clarke S, Diaz-Rubio E, Scheithauer W, Figer A, Wong R, Koski S, Sirzen F, Gilberg F, Saltz L (2008b) XELOX vs FOLFOX4: update of efficacy results from XELOX-1/NO16966, a randomized phase III trial of first-line treatment for patients (pts) with metastatic colorectal cancer (MCRC). Presented at the ASCO GI Cancers Symposium: Orlando, Florida, USA, 25–27 January (abstract 341)

[bib4] Chu E, Schulman KL, Zelt S (2009) Costs associated with complications are lower with capecitabine than with 5-fluorouracil in patients with colorectal cancer. Cancer 115: 1412–1423 19195048 10.1002/cncr.24131

[bib5] Comella P, Massidda B, Filippelli G, Farris A, Natale D, Barberis G, Maiorino L, Palmeri S, Condemi G, Southern Italy Cooperative Oncology Group (2009) Randomised trial comparing biweekly oxaliplatin plus oral capecitabine versus oxaliplatin plus i.v. bolus fluorouracil/leucovorin in metastatic colorectal cancer patients: results of the Southern Italy Cooperative Oncology study 0401. J Cancer Res Clin Oncol 135: 217–226 18719941 10.1007/s00432-008-0454-7 PMC12160290

[bib6] Cuppone F, Bria E, Sperduti I, Di Maio M, Carlini P, Milella M, Cognetti F, Terzoli E, Giannarelli D (2008) Capecitabine (CAP) versus 5-fluorouracil (FU) in combination with oxaliplatin (OX) as 1st-line chemotherapy (CT) for advanced colorectal cancer (ACRC): meta-analysis of randomized clinical trials (RCT). J Clin Oncol 26: 192s (Suppl; abstract 4056)

[bib7] de Gramont A, Figer A, Seymour M, Homerin M, Hmissi A, Cassidy J, Boni C, Cortes-Funes H, Cervantes A, Freyer G, Papamichael D, Le Bail N, Louvet C, Hendler D, de Braud F, Wilson C, Morvan F, Bonetti A (2000) Leucovorin and fluorouracil with or without oxaliplatin as first-line treatment in advanced colorectal cancer. J Clin Oncol 18: 2938–2947 10944126 10.1200/JCO.2000.18.16.2938

[bib8] Díaz-Rubio E, Tabernero J, Gómez-España A, Massutí B, Sastre J, Chaves M, Abad A, Carrato A, Queralt B, Reina JJ, Maurel J, González-Flores E, Aparicio J, Rivera F, Losa F, Aranda E, Spanish Cooperative Group for the Treatment of Digestive Tumors Trial (2007) Phase III study of capecitabine plus oxaliplatin versus continuous infusion fluorouracil plus oxaliplatin as first-line therapy in metastatic colorectal cancer: final report of the Spanish Cooperative Group for the Treatment of Digestive Tumors trial. J Clin Oncol 25: 4224–4230 17548839 10.1200/JCO.2006.09.8467

[bib9] Di Leo A, Buyse M, Bleiberg H (2004) Is overall survival a realistic primary end point in advanced colorectal cancer studies? A critical assessment based on four clinical trials comparing fluorouracil plus leucovorin with the same treatment combined either with oxaliplatin or with CPT-11. Ann Oncol 15: 545–549 15033657 10.1093/annonc/mdh127

[bib10] Ducreux M, Bennouna J, Hebbar M, Ychou M, Lledo G, Conroy T, Adenis A, Faroux R, Rebischung C, Bergougnoux L, Kockler L, Douillard JY, GI Group of the French Anti-Cancer Centers (2011) Capecitabine plus oxaliplatin (XELOX) versus 5-fluorouracil/leucovorin plus oxaliplatin (FOLFOX-6) as first-line treatment for metastatic colorectal cancer. Int J Cancer 128: 682–690 20473862 10.1002/ijc.25369

[bib11] Garrison L, Cassidy J, Saleh M, Lee F, Mena R, Fuloria J, Chang V, Ervin T, Stella P, Saltz L (2007) Cost comparison of XELOX compared to FOLFOX4 with or without bevacizumab (bev) in metastatic colorectal cancer. J Clin Oncol 25: 18S (Suppl, abstract 4074)

[bib12] Hoff PM, Ansari R, Batist G, Cox J, Kocha W, Kuperminc M, Maroun J, Walde D, Weaver C, Harrison E, Burger HU, Osterwalder B, Wong AO, Wong R (2001) Comparison of oral capecitabine versus intravenous fluorouracil plus leucovorin as first-line treatment in 605 patients with metastatic colorectal cancer: results of a randomized phase III study. J Clin Oncol 19: 2282–2292 11304782 10.1200/JCO.2001.19.8.2282

[bib13] Porschen R, Arkenau H-T, Kubicka S, Greil R, Seufferlein T, Freier W, Kretzschmar A, Graeven U, Grothey A, Hinke A, Schmiegel W, Schmoll HJ, AIO Colorectal Study Group (2007) Phase III study of capecitabine plus oxaliplatin compared with fluorouracil and leucovorin plus oxaliplatin in metastatic colorectal cancer: a final report of the AIO Colorectal Study Group. J Clin Oncol 25: 4217–4223 17548840 10.1200/JCO.2006.09.2684

[bib14] Rothenberg ML, Cox JV, Butts C, Navarro M, Bang YJ, Goel R, Gollins S, Siu LL, Laguerre S, Cunningham D (2008) Capecitabine plus oxaliplatin (XELOX) versus 5-fluorouracil/folinic acid plus oxaliplatin (FOLFOX-4) as second-line therapy in metastatic colorectal cancer: a randomized phase III noninferiority study. Ann Oncol 19: 1720–1726 18550577 10.1093/annonc/mdn370

[bib15] Saltz L, Clarke S, Díaz-Rubio E, Scheithauer W, Figer A, Wong R, Koski S, Lichinitser M, Yang TS, Rivera F, Couture F, Sirzén F, Cassidy J (2008) Efficacy and safety of bevacizumab in combination with oxaliplatin-based chemotherapy as first-line therapy in metastatic colorectal cancer: a randomized phase III study. J Clin Oncol 26: 2013–2019 18421054 10.1200/JCO.2007.14.9930

[bib16] Scheithauer W, Cassidy J, Figer A, Wong R, Koski S, Lichinitser M, Yang T, Clarke S, Diaz-Rubio E, Garrison L (2007) A comparison of medical resource use for 4 chemotherapy regimens as first-line treatment for metastatic colorectal cancer (MCRC): XELOX vs. FOLFOX4 ± bevacizumab (A). J Clin Oncol 25: 18S (Suppl, abstract 4098)

[bib17] Twelves C, Wong A, Nowacki MP, Abt M, Burris III H, Carrato A, Cassidy J, Cervantes A, Fagerberg J, Georgoulias V, Husseini F, Jodrell D, Koralewski P, Kröning H, Maroun J, Marschner N, McKendrick J, Pawlicki M, Rosso R, Schüller J, Seitz JF, Stabuc B, Tujakowski J, Van Hazel G, Zaluski J, Scheithauer W (2005) Capecitabine as adjuvant treatment for stage III colon cancer. N Engl J Med 352: 2696–2704 15987918 10.1056/NEJMoa043116

[bib18] Van Cutsem E, Twelves C, Cassidy J, Allman D, Bajetta E, Boyer M, Bugat R, Findlay M, Frings S, Jahn M, McKendrick J, Osterwalder B, Perez-Manga G, Rosso R, Rougier P, Schmiegel WH, Seitz JF, Thompson P, Vieitez JM, Weitzel C, Harper P, Xeloda Colorectal Cancer Study Group (2001) Oral capecitabine compared with intravenous fluorouracil plus leucovorin in patients with metastatic colorectal cancer: results of a large phase III study. J Clin Oncol 19: 4097–4106 11689577 10.1200/JCO.2001.19.21.4097

